# Amyloids of α-Synuclein Promote Chemical Transformations of Neuronal Cell Metabolites

**DOI:** 10.3390/ijms241612849

**Published:** 2023-08-16

**Authors:** Istvan Horvath, Khadra A. Mohamed, Ranjeet Kumar, Pernilla Wittung-Stafshede

**Affiliations:** Department of Life Sciences, Chalmers University of Technology, 412 96 Gothenburg, Sweden

**Keywords:** Parkinson’s disease, α-synuclein, amyloid, metabolomics, LC-MS

## Abstract

The assembly of α-synuclein into cross-β structured amyloid fibers results in Lewy body deposits and neuronal degeneration in Parkinson’s disease patients. As the cell environment is highly crowded, interactions between the formed amyloid fibers and a range of biomolecules can occur in cells. Although amyloid fibers are considered chemically inert species, recent in vitro work using model substrates has shown α-synuclein amyloids, but not monomers, to catalyze the hydrolysis of ester and phosphoester bonds. To search for putative catalytic activity of α-synuclein amyloids on biologically relevant metabolites, we here incubated α-synuclein amyloids with neuronal SH-SY5Y cell lysates devoid of proteins. LC-MS-based metabolomic (principal component and univariate) analysis unraveled distinct changes in several metabolite levels upon amyloid (but not monomer) incubation. Of 63 metabolites identified, the amounts of four increased (3-hydroxycapric acid, 2-pyrocatechuic acid, adenosine, and NAD), and the amounts of seventeen decreased (including aromatic and apolar amino acids, metabolites in the TCA cycle, keto acids) in the presence of α-synuclein amyloids. Many of these metabolite changes match what has been reported previously in Parkinson’s disease patients and animal–model metabolomics studies. Chemical reactivity of α-synuclein amyloids may be a new gain-of-function that alters the metabolite composition in cells and, thereby, modulates disease progression.

## 1. Introduction

Amyloid fibrils are polymers of monomeric protein units that are non-covalently assembled through β-strands and arranged perpendicularly to the fibril axis, forming a cross-β structure [1]. Many proteins can form amyloid fibrils at certain solvent conditions in vitro [1]; however, this process is mostly connected to human neurodegenerative diseases, such as Alzheimer’s disease and Parkinson’s disease (PD) [2,3,4]. In these diseases, the amyloid fibrils are often considered chemically inert end products with intermediate species (so-called oligomers) formed during aggregation as the most toxic species to cells. Nonetheless, the amyloid fibers are toxic, too, as they can seed the formation of more amyloids and transfer from cell to cell. Deleterious gain-of-functions coupled with the process of amyloid formation include mitochondrial dysfunction, oxidative stress, protein degradation failure, and, eventually, cell death [5]. We here explore the idea that catalytic activity is a new functionality of amyloid fibers.

PD is the second most common neurodegenerative disorder after Alzheimer’s disease [6,7]. The hallmark of PD is pathological intraneuronal inclusions, Lewy bodies, that contain α-synuclein amyloids [8,9,10,11]. In accordance with being the key amyloidogenic protein in PD, multiplications, and point-mutations in the α-synuclein gene, enhancing protein expression and aggregation, are linked to familial PD cases. α-synuclein amyloid fibrils can be transmitted from cell to cell, cross the blood–brain barrier, and can trigger the further transformation of monomeric α-synuclein to amyloids via elongation and secondary nucleation processes [12]. We recently reported the surprising observation that α-synuclein amyloid fibers exhibit esterase and phosphatase activity in vitro [13]. Catalytic activity on similar ester and phosphoester model substrates in vitro for amyloid-β (Alzheimer’s peptide) and glucagon (peptide hormone regulating blood glucose levels) amyloids has also been reported [14,15]. Thus, catalytic activity is a putative new functionality of human disease-related amyloids. Although amyloid structures have been exploited in research on nanomaterials, for example, by engineering short synthetic amyloid-forming peptides to incorporate catalytic active sites [16,17,18,19,20,21,22], the idea that natural amyloids harbor intrinsic catalytic activity has not been explored (except for the three in vitro studies mentioned above).

Studies in animal models of PD, PD patients’ tissue and body fluids, as well as cell culture systems challenged with α-synuclein amyloids have shown alterations in many metabolic pathways and individual metabolites [23,24]. Increased lipid and fatty acid metabolism, have been identified in human PD samples as well as in PD animal and cell models [25,26,27,28]. Energy metabolism was affected in multiple studies, with significant decreases in the levels of metabolites in the TCA cycle [26,28,29]. Additionally, purine metabolism (including uric acid, hypoxanthine, and inosine) was found to be affected in PD and PD-like conditions [30,31,32]. Decreased levels of branched-chain amino acids, including glutamine and aromatic amino acids, have also been noted in vitro and ex vivo studies of PD [33,34,35,36]. Although many of these metabolite changes might be indirect consequences of the protein aggregation process, we speculate that some may, in fact, be due to catalytic activity of the amyloids themselves.

To specifically assess catalytic activity of α-synuclein amyloid fibers on biological substrates, we here performed activity-based metabolomic experiments [37]. For this, we incubated the protein-free cell lysate of neuroblastoma cells with α-synuclein amyloid fibers and analyzed the affected metabolites via LC-MS metabolomic profiling. Of 63 identified metabolites, we found four that increased and seventeen that decreased upon α-synuclein amyloid incubation.

## 2. Results

**Figure 1:**
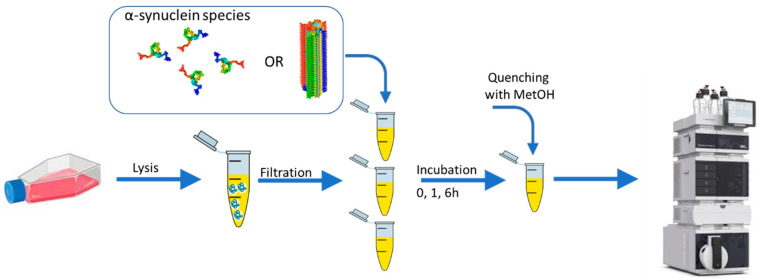
Sample preparation scheme for LC-MS metabolomic analysis. Neuronal cell lysate samples (with proteins and larger molecules removed) were treated with 20 µM freshly gel-filtered monomeric α-synuclein or pre-formed amyloids for 0 (no incubation, control), 1, and 6 h. Methanol (MetOH) was used to precipitate α-synuclein and, thus, quench any ongoing reactions. This also facilitated the removal of the protein by centrifugation prior to LC-MS analysis of the resulting metabolites.

Protein-free SH-SY5Y cell lysate samples were prepared, as described in the Materials and Methods section, and are illustrated schematically in Figure 1. The initial α-synuclein amyloid fibers were prepared in a two-step process to attain high sample homogeneity. The same amyloid stock was used to seed fresh monomers into amyloids for each biological replica. All amyloids were characterized by AFM and CD, confirming the presence of typical amyloids with a high β-sheet secondary structure content (Figure S1). Amyloid (centrifuged just before addition to remove any minor fractions of monomers and oligomers), monomer, and buffer-only treated neuronal-cell lysate samples were incubated for 0, 1, and 6 h, followed by the quenching of reactions and α-synuclein removal via methanol precipitation. The resulting samples were submitted for LC-MS analysis. Using an untargeted approach, 2000 mass features were detected in the samples. The targeted pre-processing of the peaks using in-house standards (library of known serum, plasma, and human cell line metabolites; https://www.swedishmetabolomicscentre.se (accessed 15 June 2023) yielded 63 identified metabolites (Table S1).

**Figure 2:**
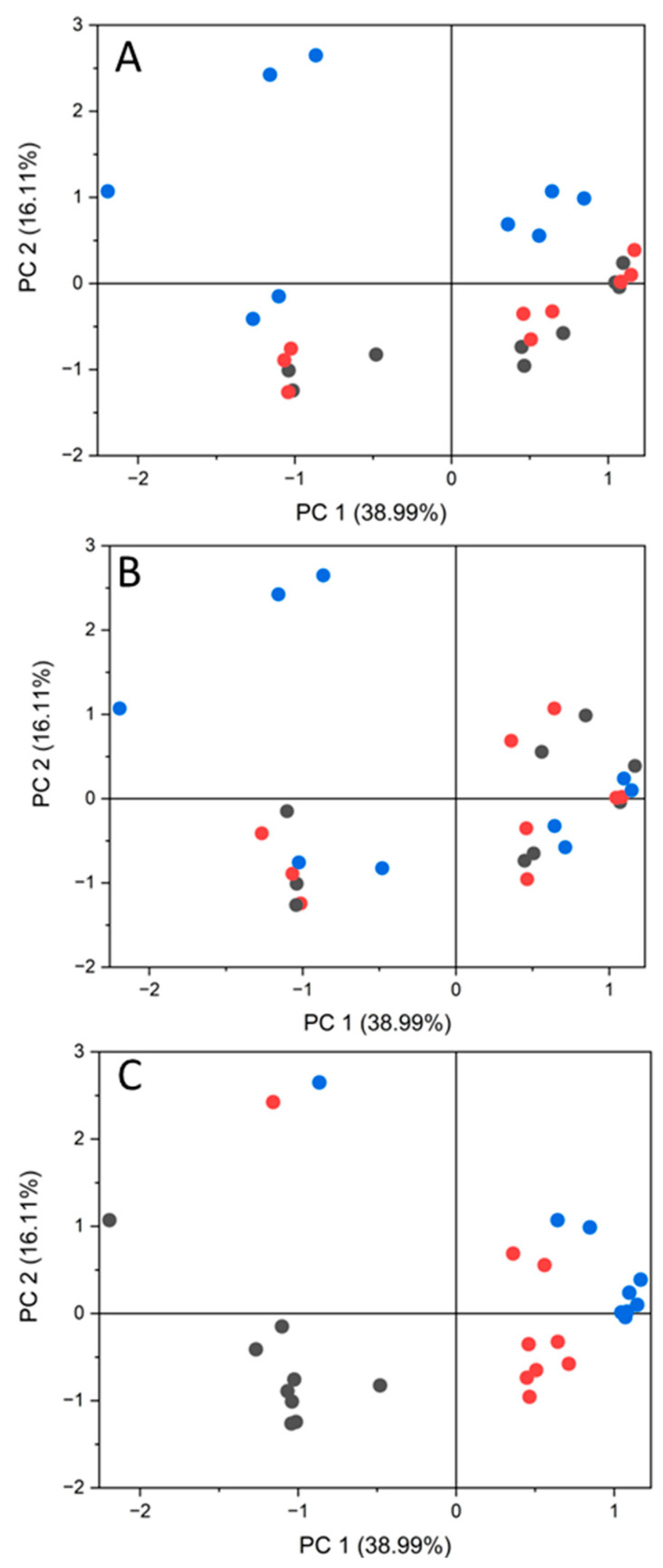
Multivariate PCA of the 63 identified metabolites with coloring according to (**A**) Treatment: black, lysate + buffer; red, lysate + monomer; blue, lysate + fiber, (**B**) Incubation time: black, 0 h; red, 1 h; blue, 6 h, and (**C**) Biological replicate (lysate batch): black, batch 1; red, batch 2; blue, batch 3.

To obtain an overview of the results, principal component analysis (PCA) was performed on data for the 63 identified metabolites (Figure 2). The PCA analysis uncovered a high variability between the three biological replicates, indicating that different batches of lysate preparations had different metabolite levels (Figure 2C). This variability between lysates might explain why no more than 63 metabolites were identified in all the samples. There were clear differences between the treatments (Figure 2A) and the incubation times (Figure 2B) with amyloid fiber treatment, especially at 6 h, which was clearly separated from the others in the PCA plot. Notably, monomer treatment, buffer treatment, and untreated lysate had very similar PCA profiles (Figure 2 and Figure S2).

Due to the variability of metabolite levels between the biological replicates, the univariate analysis of the data was performed on abundance values normalized to the 0 h time point (Materials and Methods). The individual analysis of the data for the 63 metabolites revealed significant differences (at 1 and 6 h) in the metabolite level between amyloid and monomer-treated samples for 21 of the metabolites. Table 1 lists the names of these metabolites along with the fold change in metabolite level when comparing amyloid versus monomer-incubated samples. We found that the abundance increased for four metabolites and decreased for seventeen in amyloid-treated samples. Notably, all but one of the remaining 42 metabolites showed no change upon incubation with either α-synuclein amyloids or monomers (Figures S3 and S4). For one metabolite, its level decreased upon both monomer and amyloid incubation (as well as with a buffer addition, too); this result was dismissed as an artifact caused by contamination in the lysate.

For the 21 metabolites with significant differences between the monomer and amyloid incubation, it is important to emphasize that the abundance changed in amyloid-treated samples but remained constant in monomer-treated samples. These observations strongly suggest that the presence of amyloids promotes chemical transformations that result in more or less certain metabolites.

**Figure 3:**
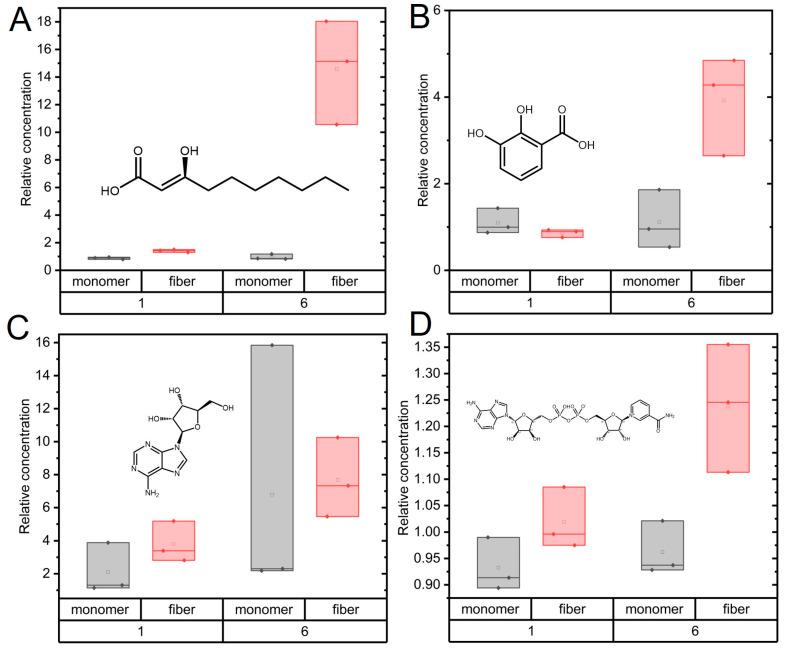
Time-dependent changes (1 and 6 h as compared to 0 h) of metabolites which showed increased levels with amyloid fiber treatment compared to monomer treatment. α-synuclein fibers (red) or monomers (black) treatments are shown as box plots. (**A**) 3-hydroxycapric acid; (**B**) 2-pyrocatechuic acid/2,4 dihydroxybenzoic acid; (**C**) adenosine; (**D**) NAD. The boxes cover the full range of the data; the line denotes the median. Chemical structures of metabolites are shown as insets.

Metabolites that were found to be increased (Figure 3) were 3-hydroxycapric acid (a fatty acid), 2-pyrocatechuic acid (related to oxidative stress [38]), adenosine (nucleobase, degradation product of ATP), and NAD (crucial cofactor in many metabolic pathways). One could speculate that the two acids are produced from ester hydrolysis (α-synuclein amyloid activity found in vitro) of parent metabolites; adenosine and NAD could be formed via the dephosphorylation (α-synuclein amyloid activity found in vitro) of AMP and NADP, respectively. In vitro experiments that assess these possible reaction mechanisms one by one are currently in development.

**Figure 4:**
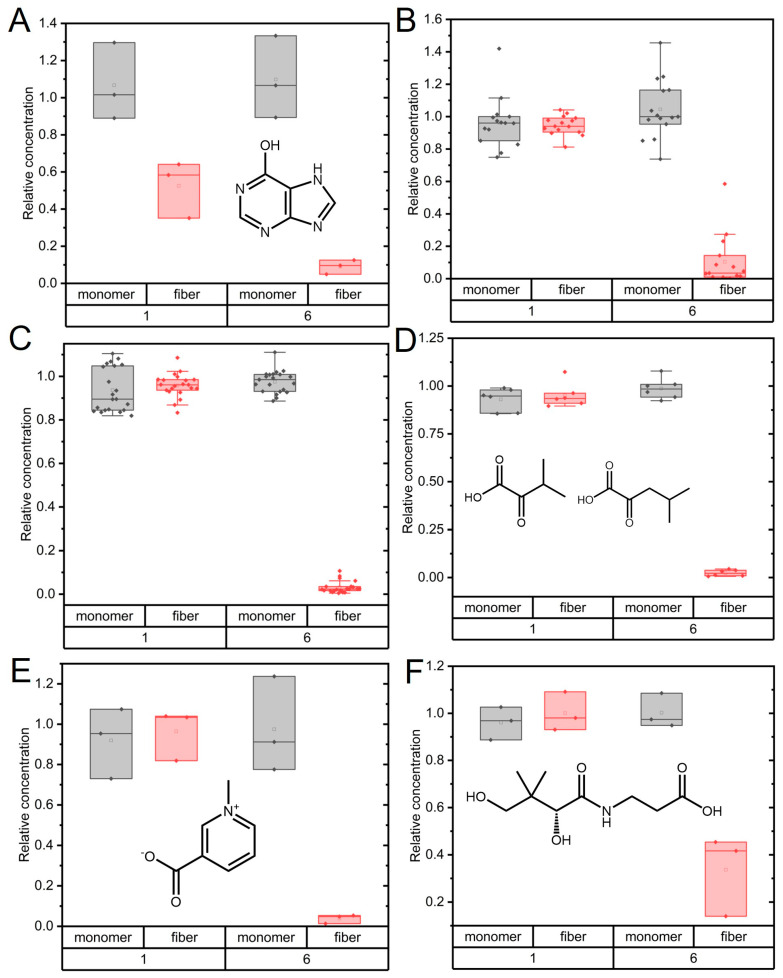
Time-dependent changes in metabolites which showed decreased levels with amyloid fiber treatment compared to monomer treatment. α-synuclein fiber (red) or monomer (black) treatments are shown as box plots. (**A**) Hypoxanthine; (**B**) Merged data for TCA cycle metabolites (oxoglutaric acid, succinic acid, malic acid, citric acid, and L-2-hydroxyglutaric acid/3-hydroxiglutaric acid); (**C**) Merged data for amino acids (Phe, Tyr, Ile, Leu, Ac-Ala, and Ac-Ser); (**D**) Merged data for α-ketoisovaleraic acid and ketoleucine; (**E**) Trigonelline; (**F**) Pantothenic acid (vitamin b5). For (**A**,**E**,**F**), boxes cover the full range of measured data, while for (**B**–**D**), the boxes cover the 25–75% range of the data. The line denotes the median. Chemical structures of some metabolites are shown as insets.

Most affected metabolites (17) showed decreased abundance upon incubation with α-synuclein amyloids (Figure 4). Thus, these molecules were converted to something else in the presence of α-synuclein amyloids but not in the presence of α-synuclein monomers. In this group of metabolites, we found hypoxanthine, which is an intermediate of the uric acid cycle [31]. Unfortunately, the two downstream metabolites of hypoxanthine (xanthine and uric acid) were not identified in the targeted analysis. Additionally, several metabolites involved in the TCA cycle (fatty acids) showed decreased levels upon α-synuclein amyloid treatment. Since α-synuclein is not a mitochondrial protein, interactions with these metabolites would only occur in vivo if the mitochondrial membrane was damaged.

Another group of compounds that dramatically decreased in abundance upon amyloid treatment for 6 h was apolar and aromatic amino acids. A potential degradation reaction might be trans-amination or de-amination, which would result in increased levels of keto-acids. However, the few keto acids we identified in this work (four) all showed decreased abundance (Table 1). Another possible route is decarboxylation, which results in amino acids being converted to amines. The only amine detected in our dataset was serotonin (created upon Trp oxidation and decarboxylation), and it had unchanged levels during the incubation (Figures S3F and S4F). Since both amino acids and keto acids decreased, it is possible that they all underwent amyloid-mediated decarboxylation. Importantly, the depletion of tyrosine and/or phenylalanine will perturb dopamine synthesis. Both amino acids are precursors of dopamine, and the decreased level of this neurotransmitter is a hallmark of PD [39]. We also identified the decreased abundance of trigonelline and pantothenic acid (vitamin b5) in amyloid fiber-treated samples after 6 h (Figure 4). Trigonelline is a crucial metabolite in NAD synthesis, and pantothenic acid is required for coenzyme-A synthesis.

We note that the disappearance of metabolites may also be due to direct binding (but not chemical conversion) to the amyloids. If this took place, these compounds would be removed from the samples together with the α-synuclein prior to LC-MS analysis. Although many small molecules have been reported to bind to α-synuclein amyloids [40], it is unlikely that all 17 metabolites we found to decrease would simply bind to the amyloids. However, it might be the case for some apolar amino acids as they could display an affinity towards hydrophobic regions of the amyloids. Still, pure amyloid binding of metabolites should have been detected already at 1 h of incubation. This was not observed: most metabolite decreases were only detected at the 6 h time point (Figure 4).

## 3. Discussion

Recent in vitro studies by us and others on amyloid catalytic activity [13,14,15] challenge the concept of amyloids as chemically inert species. Due to the repetitive in-register packing of identical polypeptides on top of each other in the folded core of amyloids, distinct arrays of hydrophilic amino acids (stacked on top of each other) might exist that form exposed catalytic sites all along the amyloid fiber surface [41]. The arrangement of possible clusters of hydrophilic residues on the amyloid surface will vary depending on what amyloidogenic protein forms the amyloid and the exact amyloid polymorph of that protein. Amyloids of α-synuclein have been shown to adopt many different folds depending on the solution conditions and mutations [42]. Despite this variability in amyloid folds, all amyloid structures will have some exposed repetitive arrays of hydrophilic residues that may become poised toward catalytic activity via the amyloid scaffold. Small molecules have been reported to bind to specific sites in α-synuclein amyloids when added in high concentrations [40]. The sole histidine residue (His50) in α-synuclein was found to be crucial for dephosphorylation activity of α-synuclein amyloids in vitro [13].

The three previous studies on amyloid catalysis were limited to studies on model substrates in test tubes. To search for the biological relevance of amyloid catalytic activity, and to identify new chemical reactions and putative substrates, here, we used a neuronal cell lysate as the pool of substrates. Our metabolic profiling results clearly show α-synuclein amyloids to harbor distinct effects on the metabolites not found for monomer or buffer treatments. In the presence of amyloids for 6 h, we found four metabolites to increase in amount and seventeen metabolites to decrease in amount. For these 21 metabolites, there was no change in their amount upon incubation with α-synuclein monomers. Even if we cannot claim catalysis, as we do not know the absolute concentrations of the metabolites, this result clearly demonstrates that, in the presence of amyloids, chemical transformations of neuronal cell metabolites take place. Unfortunately, no substrate–product pairs could be identified. Thus, we could only make speculations about involved chemical transformations. Future work should test these speculations in vitro one by one, for example, using labeled substrates that are tailor-made for each proposed reaction (using, for example, the strategy in [40]). In addition, one may develop a targeted metabolomics approach (based on the initial results presented here) to specifically search for substrate–product pairs.

As noted in the introduction, metabolic changes have been detected in samples from PD patients [43] and cell culture experiments, where cells have been challenged with amyloidogenic proteins, have shown alterations in many metabolites [27,43,44]. The reported metabolite alterations in those more complex systems largely match what we found here upon simple incubation of α-synuclein amyloids with metabolites from neuronal cell lysates. Further metabolomics studies (for example, expanding the reference library to identify more metabolites and studying the effects on lysate metabolites by pathological α-synuclein mutant amyloids) as well as more quantitative in vitro experiments (for example, assessing possible decarboxylation activity, aromatic amino acid binding, as well as reactivity toward lipids, the latter are crucial biomolecules that we did not assess here) are warranted. Taken together, the presented results underscore the possibility of yet unexplored chemical reactivity of amyloid fibers in neurodegenerative disorders (such as PD) that may contribute to disease progression.

## 4. Materials and Methods

Protein preparation: α-synuclein was expressed and purified from *E. coli* using ion exchange and gel filtration chromatography as described previously [45]. The purified protein was stored at −80 °C. The preparation of amyloid fibers was performed as described in our earlier work [13]. In short, as a first step, α-synuclein was aggregated with the help of glass bead agitation, and the formed amyloids were used as seeds in a subsequent reaction with monomeric protein at quiescent conditions. The resulting pure (and structurally homogeneous) amyloid stock was then aliquoted, flash-frozen in liquid nitrogen, and stored at −80 °C. These aliquots of amyloids were used as seeds to prepare fresh amyloid fibers from monomeric α-synuclein for each biological replica of the metabolomics experiments.

Metabolomics sample preparation: SH-SY5Y cells were seeded in 5 T75 cell culture flasks and incubated at 37 °C until 90% confluency was reached. In short, the growth medium was removed, and the cells were washed twice with pre-warmed 5 mL DPBS. Next, 5 mL of ice-cold DPBS was added to each flask, and a plastic cell scraper was used to harvest the cells. The cells were then collected in a 50 mL falcon tube. All the cells were pooled together and lysed mechanically using sonication at 30% amplitude for 9 s on/off cycles for 3 min. The lysate was then centrifuged at 18,000× *g* for 10 min at 4 °C. The supernatant was then filtered in two steps: (1) for the removal of large particles, a 0.45 μm membrane filter was used, and (2) a 3 kDa centrifugal filter was used at 15,000× *g* for 45 min at 4 °C. The purpose of the second filtration was to remove any active enzymes from the lysate. Finally, the lysate was stored at −80 °C until treatment with the protein species. The same procedure was performed with cell culture flasks without cells to generate samples that would only contain potential contamination from the culture flask and cell culture medium. Three batches of cell culture lysates were prepared and exposed to the same protein/buffer treatments.

For the next step, we added α-synuclein monomers (20 μM), amyloid fibers (20 μM in monomer units), or phosphate (PBS) buffer to the lysate samples (200 μL/sample). The amyloid sample (fresh monomers seeded by the stock amyloid sample) was first centrifuged to remove any small amount of monomers or oligomers and was then resuspended in a buffer. For this, each lysate sample was divided into 3 categories: (1) lysate + α-synuclein monomers, (2) lysate + α-synuclein amyloids, and (3) lysate + buffer. To stop reactions, ice-cold methanol was added to all the lysate samples at different time points: 0 h, 1 h, and 6 h. Centrifugation was used to remove precipitated α-synuclein protein. In addition to the three treatments, lysate samples without any additions were treated the same and used as controls. The samples were stored at −80 °C until delivery to the Swedish Metabolomics Centre. For further details, see SI Methods.

Data processing: LC-MS experimental details, data pre-processing, and identification details are described in SI Methods. The data for the 63 identified metabolites were used for PCA. Data normalization was performed by dividing the peak integrals for each metabolite with its value at t = 0 for the corresponding sample. A pair–sample t-test was used to identify significant differences between the amyloid- and monomer-treated samples using normalized data. The level of significance was set to 0.05. Data processing and analysis were performed using OriginPro 2020 software (OriginLab Corp.).

## Figures and Tables

**Table 1:** Metabolites which increased (4, Figure 3) or decreased (17, Figure 4) in amyloid-incubated samples but not in monomer-incubated samples. Fold change values were calculated based on the average relative abundance of the metabolites in amyloid vs. monomer samples at the 6 h time point. Note that the metabolites in the monomer-treated samples did not change significantly upon incubation. The metabolites were assigned to categories based on information from the human metabolome database: www.hmdb.ca (accessed on 15 June 2023).

	Metabolite	Fold Change (Amyloid vs. Monomer)
Amino compounds	L-Leucine	0.04
	N-Acetylserine	0.07
	L-Tryptophan	0.02
	N-Acetyl-L-alanine	0.02
	L-Isoleucine	0.04
	L-Tyrosine	0.02
	L-Phenylalanine	0.01
Fatty acids	3-Hydroxycapric acid	16.2
	Succinic acid	0.09
	Malic acid	0.02
	Citric acid	0.27
	L-2-Hydroxyglutaric acid/ 3-Hydroxyglutaric acid	0.04
Benzenoids	2,4-Dihydroxybenzoic acid/ 2-Pyrocatechuic acid	2.8
Nucleosides/purines	Adenosine NAD Hypoxanthine	2.1 (data for t = 1 h) 1.28 0.09
Keto acids	α-Ketoisovaleric acid	0.04
	Ketoleucine	0.01
	Pantothenic acid	0.35
	Oxoglutaric acid	0.01
Other	Trigonelline	0.04

## Data Availability

The data presented in this study is available on request.

## Supplementary Materials

The following supporting information can be downloaded at: https://www.mdpi.com/article/10.3390/ijms241612849/s1.
