# Supplementary material for: Amyloids of α-Synuclein Promote Chemical Transformations of Neuronal Cell Metabolites
Source: Int J Mol Sci. 2023 Aug 16;24(16):12849. doi: 10.3390/ijms241612849 (PMC10454467; doi:10.3390/ijms241612849)
Supplement: Supplementary file 1 [file ijms-24-12849-s001.zip › ijms-2517156-supplementary.pdf]

# SUPPLEMENTARY INFORMATION

## Amyloids of α-synculein promote chemical transformations of neuronal cell metabolites

Istvan Horvath, Khadra A. Mohamed, Ranjeet Kumar, and Pernilla Wittung-Stafshede

<u>**Content:**</u>

**Methods** description of LC-MS metabolomic analysis, CD and AFM

**Table S1**, list of all 63 identified metabolites

**Figure S1**, AFM and CD of α-synuclein amyloids used in the study

**Figure S2**, PCA plot including untreated lysate control

**Figures S3** and **S4**, examples of metabolites not changing upon incubation with α-synuclein

# Methods

**Metabolic profiling**

Metabolic profiling by LC-MS was performed at the Swedish Metabolomics Center (Umeå, Sweden) as follows: _Sample Preparation_: Prior to analysis the sample was shaken at 30 Hz for 3 minutes in a mixer mill followed by centrifugation at +4 °C, 14 000 rpm (18 620g), for 10 minutes. 300µL of the acquired supernatant was transferred to micro vials and evaporated to dryness in a speed-vac concentrator. Solvents were evaporated and the samples were stored at -80 °C until analysis. Small aliquots of the remaining supernatants were pooled and used to create quality control (QC) samples. MSMS analysis (LC-MS) was run on the QC samples for identification purposes. The samples were analyzed in batches according to a randomized run order on the LC-MS.

_LC-MS analysis_: Before LC-MS analysis the sample was re-suspended in 10 + 10 µL methanol and water containing stable isotopes internal standards (13C9-Phenylalanine, 13C3-Caffeine, D4-Cholic acid, 13C9-Caffeic Acid and salicylic acid-D6 at a final concentration of 625 pg/µL). The samples were first analyzed in positive mode followed by a switch to negative mode and a second injection of each sample was performed.

The chromatographic separation was performed on an Agilent 1290 Infinity UHPLC-system (Agilent Technologies, Waldbronn, Germany). 2 µL of each sample were injected onto an Acquity UPLC HSS T3, 2.1 x 50 mm, 1.8 µm C18 column in combination with a 2.1 mm x 5 mm, 1.8 µm VanGuard precolumn (Waters Corporation, Milford, MA, USA) held at 40 °C. The gradient elution buffers were A (H2O, 0.1 % formic acid) and B (75/25 acetonitrile:2-propanol, 0.1 % formic acid), and the flow rate was 0.5 mL min-1. The compounds were eluted with a linear gradient consisting of 0.1 - 10 % B over 2 minutes, B was increased to 99 % over 5 minutes and held at 99 % for 2 minutes; B was decreased to 0.1 % for 0.3 minutes and the flow-rate was increased to 0.8 mL min-1 for 0.5 minutes; these conditions were held for 0.9 minutes, after which the flow-rate was reduced to 0.5 mL min-1 for 0.1 minutes before the next injection.

The compounds were detected with an Agilent 6546 Q-TOF mass spectrometer equipped with a jet stream electrospray ion source operating in positive or negative ion mode. The settings were kept identical between the modes, with exception of the capillary voltage. A reference interface was connected for accurate mass measurements; the reference ions purine (4 µM) and HP-0921 (Hexakis (1H, 1H, 3H-tetrafluoropropoxy)phosphazine) (1 µM) were infused directly into the MS at a flow rate of 0.05 mL min-1 for internal calibration, and the monitored ions were purine m/z 121.05 and m/z 119.03632; HP-0921 m/z 922.0098 and m/z 966.000725 for positive and negative mode respectively. The gas temperature was set to 150°C, the drying gas flow to 8 L min-1 and the nebulizer pressure 35 psig. The sheath gas temp was set to 350°C and the sheath gas flow 11 L min-1. The capillary voltage was set to 4000 V in positive ion mode, and to 4000 V in negative ion mode. The nozzle voltage was 300 V. The fragmentor voltage was 120 V, the skimmer 65 V and the OCT 1 RF Vpp 750 V. The collision energy was set to 0 V. The m/z range was 70 - 1700, and data was collected in centroid mode with an acquisition rate of 4 scans s-1 (1977 transients/spectrum).

**Metabolite data analysis**

All data processing was performed using the Agilent Masshunter Profinder version B.10.0.2 (Agilent Technologies Inc., Santa Clara, CA, USA). The data pre- processing was performed both in a targeted and an untargeted fashion. For targeted processing, a pre-defined list of metabolites commonly found in plasma and serum were searched for in the obtained untargeted dataset using the Batch Targeted feature extraction in Masshunter Profinder. An-in-house LC-MS library built up by authentic standards run on the same system with the same chromatographic and mass-spec settings, were used for the targeted processing. The identification of the metabolites was based on MS, MSMS and retention time information.

**Atomic force microscopy (AFM)**

Solution of 100 µM α-synuclein fibers were 20 times diluted into Milli-Q water and deposited on freshly cleaved mica. After 10 min, the mica was rinsed with filtered Milli-Q water and dried under a gentle nitrogen stream. Images were recorded on an NTEGRA Prima setup (NT-MDT, Moscow, Russia) using a gold-coated single crystal silicon cantilever (NT-MDT, NSG01, spring constant of ∼5.1 N/m) and a resonance frequency of ∼180 kHz in tapping mode. 512 × 512-pixel images were acquired with a 0.5-Hz scan rate. Images were analyzed using the WSxM 5.0 software

**Circular dichroism (CD) spectroscopy**

Far-UV CD of α-synuclein (10 µM) were recorded on a Chirascan spectropolarimeter (Applied PhotoPhysics, Leatherhead, UK) in 10 mM phosphate buffer at pH 7.4 CD spectra between 250 and 190 nm were collected in a quartz cuvette (path length of 1 mm) with a bandwidth of 1 nm, a step size of 1 nm, and a scanning speed of 50 nm/min.

**Table S1.** List of the 63 identified metabolites in the LC-MS experiments. Blue color denotes decreased, orange color increased, metabolite abundance upon amyloid fiber incubation as compared to incubation with monomer or buffer. The metabolites were classified according to the human metabolome database (www.hmdb.ca). See also main **Table 1** and **Figures 3** and **4**.

| Metabolite name | Classification | Metabolite name | Classification |
|---|---|---|---|
| Bis(2-ethylhexyl)phthalate | *other* | Undecanedioic acid | *fatty acids* |
| Urocanic acid | | Hydroxycaproic acid | |
| Trigonelline | | Malic acid | |
| Niacinamide | | 2-Hydroxyhexadecanoic acid | |
| Spermine | | Suberic acid | |
| Spermidine | | Glutaric acid | |
| Rutin | | Succinic acid | |
| Oleamide | | Citric acid | |
| Serotonin | *amino compounds* | Sebacic acid | |
| Pyroglutamic acid | | 3-Hydroxycapric acid | |
| L-Phenylalanine | | Azelaic acid | |
| L-Tryptophan | | Adipic acid | |
| 4-Acetamidobutanoic acid | | Pimelic acid/3-Methyladipic acid | |
| N-Acetylvaline | | L-2-Hydroxyglutaric acid/ 3-Hydroxyglutaric acid | |
| N-Acetylserine | | Alpha-ketoisovaleric acid | *keto acids* |
| N-Acetyl-L-aspartic acid | | Ketoleucine | |
| N-Acetyl-L-alanine | | Pantothenic acid | |
| L-Tyrosine | | Oxoglutaric acid | |
| L-Isoleucine | | 5'-Methylthioadenosine | *nucleosides* |
| L-Methionine | | Adenosine | |
| L-Leucine | | Adenosine monophosphate | |
| Salicylic acid | *benzenoids* | Guanosine monophosphate | |
| 2,4-Dihydroxybenzoic acid/ 2-Pyrocatechuic acid | | NAD | |
| Valerylcarnitine | *carnitines* | Uridine 5'-monophosphate | |
| Propionylcarnitine | | Hypoxanthine | *purines* |
| L-Acetylcarnitine | | Oxidized glutathione | *short length peptides* |
| L-Carnitine | | γ-Glutamylisoleucine | |
| Isovalerylcarnitine | | N-Acetylaspartylglutamic acid | |
| 4-Trimethylammoniobutanoic acid | | Isoleucyl-Isoleucine | |
| Hexanoylcarnitine | | Phenylalanylvaline | |
| Butyrylcarnitine | | Isoleucyl-Leucine | |
| | | Ophthalmic acid | |

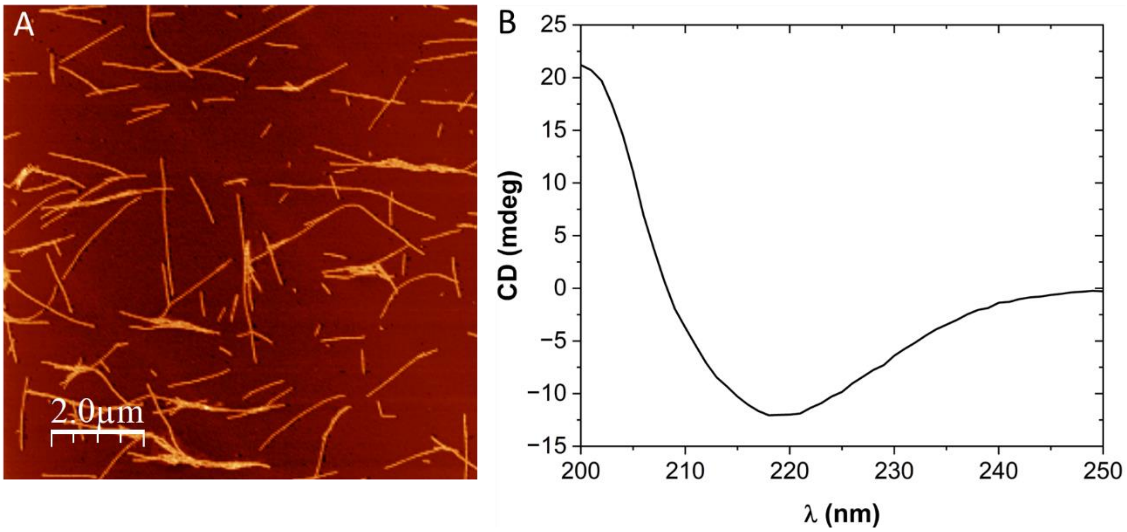


**Figure S1.** AFM image (A) and CD spectrum (B) of the α-synuclein fibers (10 µM in 10 mM phosphate, pH 7.4) used in the metabolomic studies.

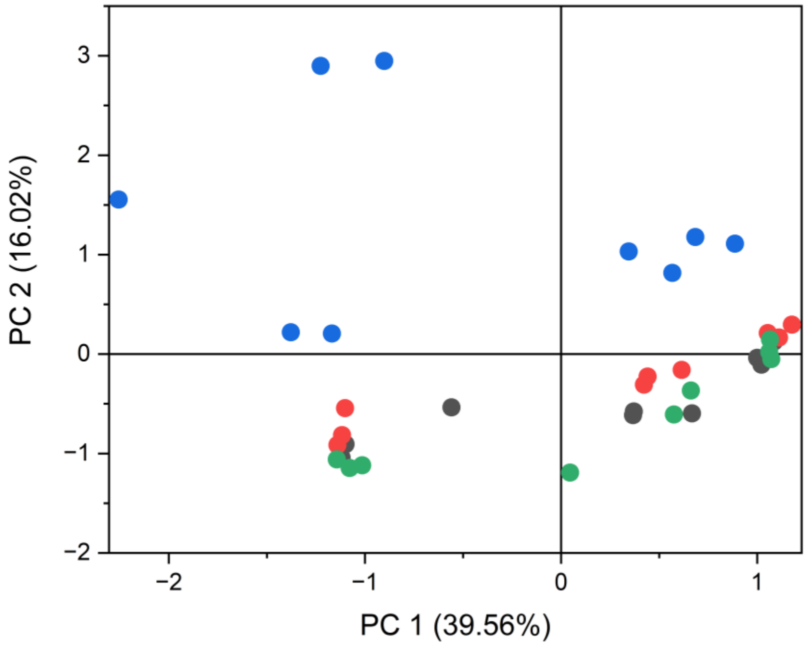


**Figure S2.** PCA of the identified 63 metabolites with coloring according to treatment type (3 time points for each, 3 biological replicas): black, lysate+buffer; red, lysate+monomer; blue, lysate+amyloid; green, untreated lysate. This is the same data as in **Figure 2**, but with the addition of untreated lysate. Note that untreated lysate, lysate+buffer and lysate+monomer all cluster together and are distinctly separated from lysate+amyloid.

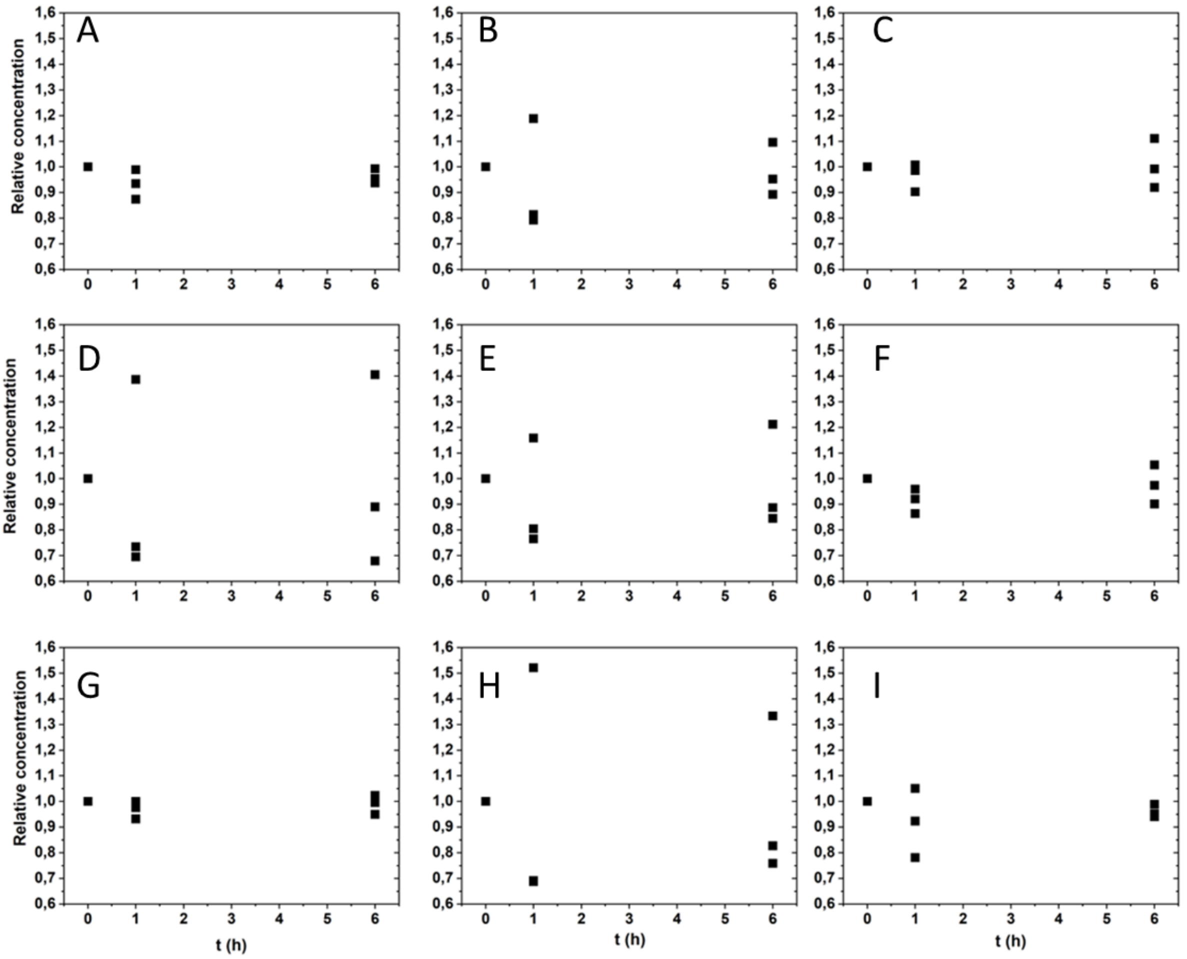


**Figure S3.** Examples of unaffected metabolites (at 1 and 6 h) in monomer treated lysate samples. (A) Adenosine monophosphate; (B) Azelaic acid; (C) Butyrylcarnitine; (D) Pimelic acid/3-Methyladipic acid; (E) Sebacic acid; (F) Serotonin; (G) Spermidine; (H) Suberic acid; (I) Valerylcarnitine. Compare to the same metabolite data for amyloid treatments in **Figure S4**.

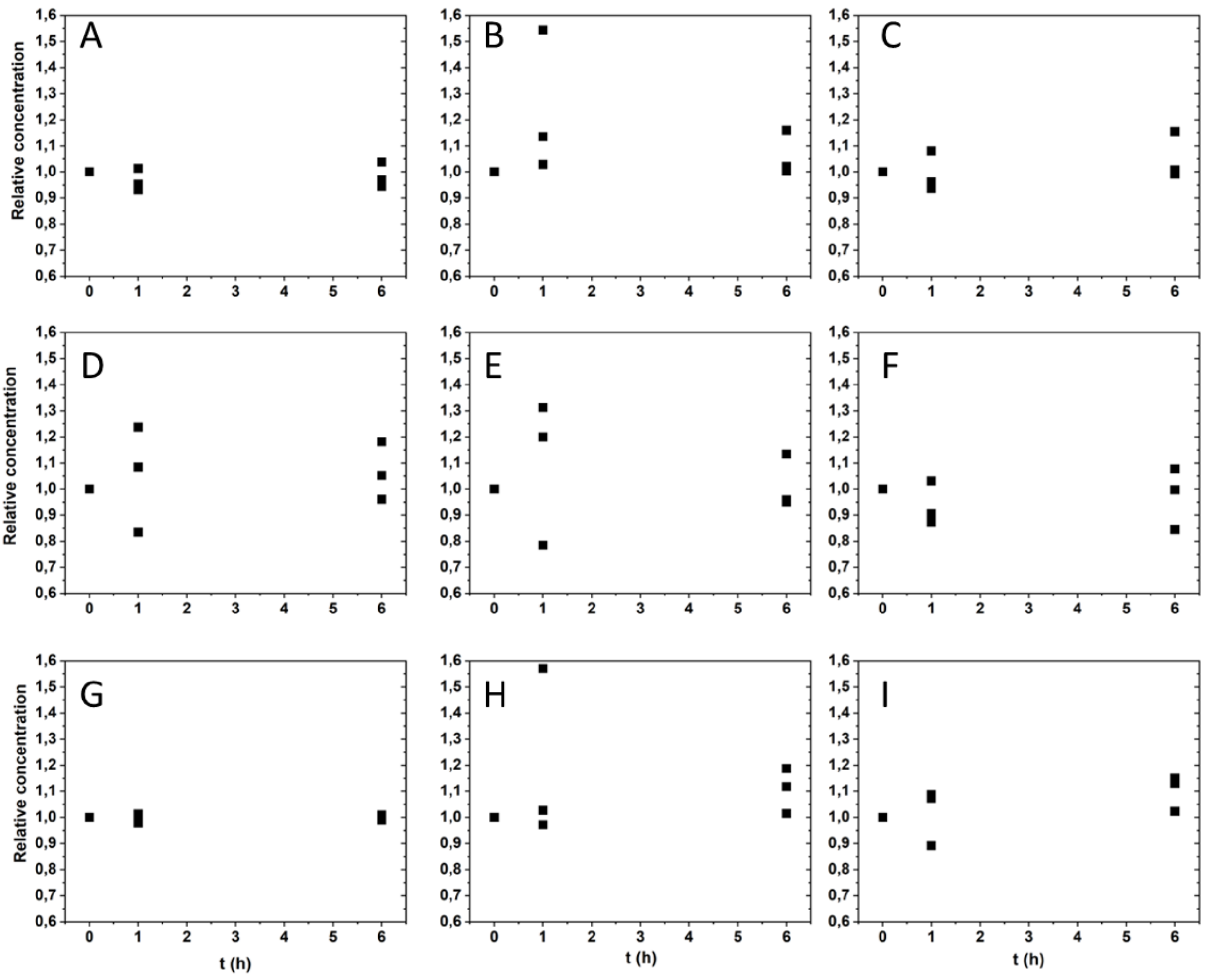


**Figure S4.** Examples of unaffected metabolites (at 1 and 6 h) in amyloid fiber treated lysate samples. (A) Adenosine monophosphate; (B) Azelaic acid; (C) Butyrylcarnitine; (D) Pimelic acid/3-Methyladipic acid; (E) Sebacic acid; (F) Serotonin; (G) Spermidine; (H) Suberic acid; (I) Valerylcarnitine. Compare to the same metabolite data for monomer treatments in **Figure S3**.
